# Pulmonary fibrosis in relation to genetic loci in an inception cohort of patients with early rheumatoid arthritis from northern Sweden

**DOI:** 10.1093/rheumatology/keab441

**Published:** 2021-05-16

**Authors:** Elias Jönsson, Lotta Ljung, Eva Norrman, Eva Freyhult, Lisbeth Ärlestig, Johanna Dahlqvist, Solbritt Rantapää-Dahlqvist

**Affiliations:** 1 Department of Public Health and Medicine/Rheumatology, Umeå University, Umeå; 2 Department of Medical Sciences, National Bioinformatics Infrastructure Sweden, Science for Life Laboratory; 3 Department of Medical Biochemistry and Microbiology, and Medical Sciences, Uppsala, University, Uppsala, Sweden

**Keywords:** rheumatoid arthritis, pulmonary fibrosis, rs35705950 (*MUC5B*), rs111521887 (*TOLLIP*), rs2609255 (*FAM13A*), rs2736100 (*TERT*)

## Abstract

**Objectives:**

Pulmonary manifestations in RA are common comorbidities. Interstitial lung disease (ILD), both idiopathic and in RA, has been associated with several genetic variants. We assessed pulmonary fibrosis (PF) in an inception cohort of RA patients in relation to genetic variants and disease-related factors.

**Methods:**

A total of 1466 early RA patients were consecutively included and followed prospectively from the index date until death or 31 December 2016. Clinical and laboratory data and treatment were continuously registered according to the Swedish Rheumatology Quality Register. DNA was available from 1184 patients and 571 151 genome-wide single-nucleotide polymorphisms (SNPs) were analysed. Thirteen identified genetic variants were extracted. At follow-up, the patients answered a questionnaire regarding disease progression and lung involvement that was validated by reviewing medical records and analysing radiological examinations.

**Results:**

The prevalence of PF was 5.6% and the annualized incidence rate was 5.0/1000 (95% CI 3.80, 6.54). Four SNPs were associated with PF in RA: rs35705950 [*MUC5B*; OR 2.5 (95% CI 1.5, 4.0), adjusted *P*-value = 0.00016, *q*-value = 0.0021]; rs111521887 [*TOLLIP*; OR 1.9 (95% CI 1.3, 2.8), adjusted *P*-value = 0.0014, *q*-value = 0.0092]; rs2609255 [*FAM13A*; OR 1.7 (95% CI 1.1, 2.5), adjusted *P*-value = 0.013, *q*-value = 0.055] and rs2736100 [*TERT*; OR 1.5 (95% CI 1.0, 2.2), adjusted *P*-value = 0.046, *q*-value = 0.15]. Older age and RF positivity were associated with increased risk, while MTX treatment was associated with a lower risk of PF.

**Conclusions:**

Development of PF in an inception cohort of RA patients was associated with 4 of 12 ILD risk genes. RA-related factors except for age at diagnosis and RF positivity were of limited importance in PF development.

Rheumatology key messages

- Development of pulmonary fibrosis in rheumatoid arthritis is associated with certain risk genes.
- Rheumatoid arthritis–related factors are of limited importance in the development of fibrosis.
- Methotrexate was not associated with increased risk of pulmonary fibrosis.

## Introduction

The lungs are a frequently involved organ in RA [1, 2]. Pulmonary involvement has been proposed as a locus of the initiation of RA and later in the disease course as a comorbidity secondary to RA or as a result of treatment complications [3, 4]. Interstitial lung disease (ILD) in RA shares several characteristics with idiopathic pulmonary fibrosis (IPF), with a progressive course and poor survival [5–8]. In particular, RA-ILD predominantly shares the imaging and pathological phenotypes of the usual interstitial pneumonia (UIP) type [6].

A strong genetic basis for IPF has been demonstrated in studies of familial aggregations [9] and in genome-wide association studies (GWASs) in the general population. The most consistent finding is of a common variant (rs35705950) in the promoter of the *MUC5B* (mucin 5B) gene [10–13], but several other genes involving host defence, cell–cell adhesion, signalling and telomere maintenance have been associated with IPF [10–12, 14, 15]. The association between the *MUC5B* promoter variant and ILD in RA patients has been confirmed with similar point estimates for risk as for IPF in the general population [16]. In addition, a study using exome sequencing has identified 13 heterozygous mutations in the coding regions of *TERT*, *RTET1*, *PAR* and *SFTPC* as associated with ILD in RA [17]. 

Predictive factors for the development of PF in RA have been reported to be similar to those for IPF and ILD in general, e.g. male sex, age and tobacco smoking [18], in addition to RA-related factors such as positive RF and/or ACPAs and disease activity [19–21].

In this study we aimed to evaluate the development of PF in relation to the previously identified gene loci for IPF in our inception cohort of patients with early RA followed prospectively within the catchment area of northern Sweden. The impact of disease-related factors was also evaluated in relation to PF.

## Materials and methods

### Subjects and collection of clinical data

An inception cohort of patients diagnosed with early RA according to the American College of Rheumatology Classification Criteria [22] were analysed in this study. The patients with early RA (symptomatic <12 months before diagnosis) were consecutively included in the study at the time of RA diagnosis (index date) between 1 January 1996 and 31 December 2016 at any of the five rheumatology clinics in northern Sweden. They were simultaneously included in the Swedish Rheumatology Quality Register (SRQ) [23]. Clinical data, e.g. the 28-joint DAS (DAS28) [24] and starting and ongoing pharmacological treatment, were registered. These data were systematically recorded at baseline and at 6, 12, 18 and 24 months after diagnosis and thereafter at all clinical visits. Data on smoking habits were collected at inclusion (the index date) and previous and current smoking was registered as smoking ever *vs* non-smoking. BMI (in m/kg^2^) was also recorded.

ACPAs were analysed in plasma samples collected at baseline (the index date) and preserved at −80° C until analysed using the anti-CCP2 test (Euro Diagnostica, Malmö, Sweden), as previously described [25]. RF was assessed at baseline using routine laboratory methods.

Radiographs of the lungs were obtained at baseline as part of the clinical routine in 95% of the cases. Thereafter, radiological examinations were performed in patients presenting any symptoms of cough, dyspnoea or chest pain and before initiating biologic DMARDs (bDMARDs). If there was any sign of pathology on the routine plain X-rays, clinically suspected pulmonary involvement or other clinical indication of ILD, high-resolution CT (HRCT) was performed. At the index date, 83 (7%) of the RA patients had a pulmonary diagnosis: asthma in 55 (4.7%), chronic obstructive pulmonary disease (COPD) in 22 (1.9%), both diagnoses in 3 and a diagnosis of bronchiectasis, chronic bronchitis or pleura plaque in 3.

In 2016 the patients completed a questionnaire regarding disease progression and the incidence of lung involvement, e.g. self-reported symptoms of cough, dyspnoea or chest pain. The answers to the questionnaires were validated by reviewing the patients’ medical records and analysing the radiological examinations of the lungs. In this study of the originally recruited RA patients with DNA collected at baseline, 1184 were eligible, although 66 patients (5.6%) declined to participate by not answering the questionnaires. Consequently they were excluded and their hospital records were not examined, resulting in 1118 patients remaining for the analysis of PF. Since their inclusion in the study, 157 patients have died and therefore did not answer the questionnaire. For these individuals, medical records were reviewed and the radiological examinations were analysed. The results of the X-rays and HRCT examinations were reassessed at the end of the follow-up by an experienced pulmonologist. The diagnosis of PF was based on the results of HRCT, with the exception of five patients for whom the PF diagnosis was based on the routine plain X-ray examinations. The HRCT findings defined as PF were reticular pattern, honeycombing or traction bronchiectasis of variable degree and with ground-glass in a few cases [26]. In this study, no further diagnostic evaluation was performed to identify and separate between UIP and non-specific interstitial pneumonia.

Pharmacological treatment was registered at the index date, after 24 months and as ever-used treatment during the study period regarding corticosteroids and conventional synthetic DMARDs (csDMARDS; MTX, SSZ, chloroquine, LEF, AZA, ciclosporin, MMF and oral or injectable gold salts). Treatment with bDMARDs (abatacept, adalimumab, anakinra, etanercept, infliximab, rituximab, tocilizumab) was recorded as prescribed during the initial 24 months and as after 24 months if ever prescribed during the study period.

### Genotyping

At inclusion, blood samples was collected and stored at −80°C for analyses (*n* = 1184). Controls [*n* = 1456; 68.1% females, mean age 51.7 years (s.d. 9.3)] for the GWAS were recruited from the Medical Biobank of Northern Sweden, a population-based biobank comprising a screening program for cardiovascular risk factors and prevention of comorbidity [27].

GWAS genotyping of DNA samples from cases and controls was performed using the Global Screen Assay (GSA; Illumina, San Diego, CA, USA) to analyse 571 151 genome-wide single-nucleotide polymorphisms (SNPs) at deCode Genetics (Reykjavík, Iceland). The genetic variations in the HLA region defined from 29.6 to 33.27 Mb in chromosome 6 were imputed using a European reference panel consisting of 5225 European individuals in the Type 1 Diabetes Genetic Consortium [28, 29]. An HLA-shared epitope was defined as 0401/0404/0405/0408 or 0101. Information for the following selected gene variants (*n* = 13) at 12 loci was extracted from the genotype data set: rs35705950 (*MUC5B*), rs2736100 (*TERT*), rs111521887 and rs5743890 (*TOLLIP*), rs2076295 (*DSP*), rs7887 (*EHMT2*), rs2034650 (*IVD*), rs2609255 (*FAM13A*), rs4727443 (*LOC100128334/LOC105375423*), rs11191865 (*OBFC1*), rs1278769 (*ATP11A*), rs6793295 (*LRRC34*) and rs12610495 (*DPP9*), according to previous publications [16].

### Pulmonary examinations

The HRCT examinations were performed during the 20 year study period at five different radiology centres, leading to modifications in the methods. The GE Medical Systems Light Speed Plus and GE Medical Revolution System (GE Healthcare, Chicago, IL, USA) were used with the following parameters: axial mode collimator total/single width (1.25/0.625 mm), rotation time 0.5 or 0.7 s, pitch/scan options at 120/140 KV; and 1.25 mm thick section at 10 mm intervals and helical mode collimator total/single width 20–40/0.625 mm, rotation time 0.5 s and 2.5–5.0 mm thick sections at 1.25–5.0 mm intervals were reconstructed with an X-ray tube current of 180–280 mAs.

### Statistical analysis

Statistical analysis was performed using R software version 3.5.0 (R Foundation for Statistical Computing, Vienna, Austria) [30] and SPSS 25.0 (IBM, Armonk, NY, USA). Descriptive data were summarized and presented as proportions, means and medians as appropriate. Frequencies were compared using the chi-squared test and for continuous data the Student’s *t* test and non-parametric tests were used. Associations between PF and possible predictors including genetic markers were analysed using logistic regression analysis and are presented as the odds ratio (OR) with the 95% CI. All multivariable models were adjusted for sex, smoking habits and age at DNA sampling (controls) or age at RA onset if not stated otherwise. The models were further adjusted for ACPA, RF, HLA-shared epitope and disease activity at the index date and during the first 24 months summarized by the calculated area under the curve for DAS28 (DAS-AUC_24_), as presented in the tables. A crude *P*-value is presented for the simple model based on the same subsets of samples as the multiple variable models for which the adjusted *P*-value is presented. The significance level was set at *P* < 0.05. A *q*-value is presented, i.e. a *P*-value adjusted for multiple testing according to the Benjamini–Hochberg false discovery rate (FDR) method; <10% was regarded as acceptable and hence the significance threshold was *q* = 0.10.

For missing data, imputations have been performed: age at sampling for controls (*n* = 34) and smoking habits for cases and controls (*n* = 75) and, for the RA patients, BMI (*n* = 197). Imputation was performed using linear/logistic regression based on age, sex, smoking and RA status (RA or control). All other data were complete except for missing data for MTX (*n* = 102).

## Results

Of the early RA patients included in our inception cohort, 60 (5.6%) had PF at the time of diagnosis or developed PF during follow-up. In two of the cases the diagnosis of PF was settled prior to the diagnosis of RA, at 5 and 7 months, respectively. In nine additional cases the diagnosis was confirmed at the same time RA was diagnosed or within 4 months of the RA diagnosis. The degree of radiological changes diagnosed as PF varied from mild to severe. The total time at risk for the patients was 10 340 person-years and the annualized incidence rate was 5.0/1000 (95% CI 3.80, 6.54) for PF. Twenty-seven patients diagnosed with PF died during the study. Demographic and clinical data of the patients stratified for PF are presented in Table 1.

**Table 1:** Demographic and clinical data of the included patients with early RA stratified for development of PF

Characteristics	Early RA with PF (*n* = 60)	Early RA without PF (*n* = 1058)
Age at RA diagnosis, mean (s.d.), years	64.8 (10.3)***	57.4 (14.1)
Age at ILD diagnosis, mean (s.d.), years	73.9 (9.6)	–
Females, *n* (%)	37 (61.7)	720 (70.7)
Ever-smoker, *n* (%)	43/59 (72.9)	623/1009 (61.7)
Current smoker, *n* (%)	11/58 (19.0)	203/1043 (19.5)
ACPA positive, *n* (%)	43 (80.0)	705 (69.2)
ACPA, median (IQR)	203.0 (547.4)	98.2 (415.0)
RF positive, *n* (%)	53 (88.3)**	739 (72.5)
HLA-shared epitope positive, *n* (%)	23 (63.9)	371 (58.7)
Follow-up time from RA diagnosis until 31 December 2016 or death, mean (s.d.), years	8.7 (4.8)	9.5 (5.0)
BMI, mean (s.d.), kg^2^/cm	27.0 (3.7)^a^	26.4 (4.5)^a^
DAS28 on index date, mean (s.d.)	4.8 (1.4)	4.8 (1.4)
DAS28-AUC_24_, mean (s.d.)	90.0 (18.8)*	82.7 (21.5)
Death during study period, *n* (%)	27 (45.0)***	130 (12.3)
Treatment at index date, *n*/*N* (%)		
Glucocorticoids	25/45 (55.6)	454/775 (58.6)
MTX	38/57 (66.7)	735/859 (85.6)***
csDMARDs ever during follow-up, *n*/*N* (%)	57/58 (98.3)	1004/1024 (98.0)
MTX ever during follow-up, *n*/*N* (%)	39/59 (66.1)	860/960 (89.6)***
bDMARDs within 24 months, *n*/*N* (%)	12/60 (20.0)*	113/1050 (10.8)
bDMARDs during follow-up, *n*/*N* (%)	23/37 (38.3)	295/1050 (28.1)
Duration of RA at time of PF diagnosis, mean (minimum–maximum), years	5.4 (−0.58–15.6)	–
HRCT, *n* (%)	55/60 (91.7)	–
VC, mean (95% CI), %^b^	88.2 (79.9, 96.5)	–
TLC, mean (95% CI), %^b^	81.3 (74.5, 88.2)	
DLCO, mean (95% CI), %^b^	61.1 (52.6, 69.7)	–

a Data available in 50 patient with PF and 849 patients without PF.

b Performed in 25 cases synchronous to radiology.

VC: vital capacity; TLC: total lung capacity; DLCO: diffusion capacity of the lungs for carbon monoxide. *P<0.05, **P<0.01, ***P<0.001.

### Associations with SNPs

#### RA patients with PF vs population controls

In the genetic association analysis, the SNPs rs35705950 (*MUC5B*) and rs111521887 (*TOLLIP*) were significantly associated with RA with PF, compared with population controls [for rs35705950: OR 4.4 (95% CI 1.9, 11.0), adjusted *P*-value = 0.00069, *q*-value = 0.009; for rs111521887: OR 2.4 (95% CI 1.2, 4.5), adjusted *P*-value = 0.0083, *q*-value = 0.054; Table 2a]. 

**Table 2:** Association between selected SNPs and RA with PF (*n* = 60) and population controls (*n* = 1292) and in RA patients with and without PF (in simple logistic regression analyses adjusted for sex, smoking habits and age at DNA sampling or age at RA onset, respectively) analysed for each SNP separately

rs Identifier	Risk allele	RA-PF *vs* controls						RA-PF *vs* RA without PF					
		MAF (RA/C)	OR (95% CI)	Crude *P*-value	OR (95% CI)	Adjusted *P*-value	*q*-Value	MAF	OR (95% CI)	Crude *P*-value (ILD/no ILD)	OR (95% CI)	Adjusted *P*-value	*q*-Value
rs35705950	T	0.26/0.12	2.39 (1.48, 3.76)	0.00021	4.41 (1.88, 10.66)	0.00069	0.009	0.26/0.11	2.57 (1.58, 4.10)	8.9e-05	2.52 (1.54, 4.04)	0.00016	0.0021
rs111521887	G	0.33/0.19	2.09 (1.40, 3.08)	0.00025	2.35 (1.25, 4.48)	0.0083	0.054	0.33/0.20	1.91 (1.28, 2.80)	0.0011	1.89 (1.27, 2.78)	0.0014	0.0092
rs2609255	G	0.33/0.24	1.58 (1.07, 2.30)	0.018	1.34 (0.72, 2.49)	0.35	0.65	0.33/0.23	1.69 (1.13, 2.50)	0.0096	1.67 (1.11, 2.49)	0.013	0.055
rs7887	T	0.47/0.37	1.55 (1.07, 2.23)	0.019	1.54 (0.85, 2.79)	0.15	0.43	0.47/0.42	1.23 (0.85, 1.79)	0.27	1.21 (0.83, 1.77)	0.32	0.59
rs2736100	A	0.61/0.55	1.25 (0.86, 1.83)	0.24	1.60 (0.91, 2.93)	0.11	0.43	0.61/0.53	1.39 (0.96, 2.03)	0.085	1.47 (1.01, 2.15)	0.046	0.15
rs12610495	G	0.43/0.38	1.23 (0.84, 1.77)	0.28	0.98 (0.51, 1.83)	0.94	0.94	0.43/0.36	1.35 (0.92, 1.96)	0.12	1.31 (0.89, 1.92)	0.16	0.42
rs2076295	G	0.47/0.41	1.29 (0.91, 1.85)	0.15	1.14 (0.64, 2.06)	0.65	0.84	0.47/0.42	1.25 (0.86, 1.82)	0.25	1.25 (0.86, 1.83)	0.24	0.53
rs5743890	C	0.08/0.10	0.85 (0.41, 1.56)	0.62	0.95 (0.34, 2.45)	0.92	0.94	0.08/0.11	0.75 (0.36, 1.39)	0.40	0.75 (0.36, 1.40)	0.41	0.66
rs4727443	C	0.38/0.36	1.10 (0.76, 1.59)	0.61	0.68 (0.36, 1.35)	0.23	0.50	0.38/0.36	1.08 (0.74, 1.58)	0.68	1.07 (0.73, 1.56)	0.73	0.93
rs1278769	G	0.22/0.21	1.04 (0.66, 1.60)	0.86	1.21 (0.60, 2.37)	0.59	0.84	0.22/0.24	0.89 (0.57, 1.46)	0.61	0.93 (0.58, 1.42)	0.73	0.93
rs11191865	A	0.44/0.47	0.90 (0.63, 1.29)	0.58	0.66 (0.36, 1.18)	0.17	0.43	0.44/0.46	0.91 (0.63, 1.32)	0.62	0.95 (0.65, 1.38)	0.79	0.93
rs6793295	C	0.32/0.29	1.12 (0.75, 1.65)	0.56	1.04 (0.55, 1.93)	0.89	0.94	0.32/031	1.01 (0.68, 1.49)	0.95	1.04 (0.69, 1.53)	0.86	0.93
rs2034650	A	0.50/0.46	1.20 (0.83, 1.73)	0.34	1.17 (0.65, 2.11)	0.60	0.84	0.50/0.49	1.02 (0.70, 1.49)	0.90	1.01 (0.69, 1.47)	0.98	0.98

#### RA patients with PF vs RA patients without PF

In logistic regression analyses, the same two SNPs, rs35705950 and rs111521887, were also associated with an increased risk of PF in RA [for rs35705950: OR 2.5 (95% CI 1.5, 4.0), adjusted *P*-value = 0.00016, *q*-value = 0.0021; for rs111521887: OR 1.9 (95% CI 1.3, 2.8), adjusted *P*-value = 0.0014, *q*-value = 0.0092; Table 2b], as was SNP rs2609255 (*FAM13A* gene locus; OR 1.7 (95% CI 1.1, 2.5), adjusted *P*-value = 0.013, *q*-value = 0.055]. The SNP rs2736100 (*TERT* gene locus) was associated with the risk of PF after adjustments [OR 1.5 (95% CI 1.0, 2.2), adjusted *P*-value = 0.046, *q*-value = 0.15] (Table 2b). Further adjustments for ACPA, RF and HLA-shared epitope did not change the associations between the SNPs; rs35705950, rs111521887 and rs2609255 and the risk of RA-PF showed similar ORs and *P*-values (Table 3). The addition of DAS28 at the index date to the mentioned adjustments did not change the ORs or the *P*-values (Table 3) and additional adjustments for BMI and MTX at the index date, included separately or together, yielded similar significant associations of the SNPs with the development of PF (data not shown).

**Table 3:** Association between RA with and without PF analysed in simple logistic regression analyses for each SNP with adjustments

rs Identifier	Risk allele	RA with vs. without PF with adjustments^a^						RA with vs. without PF with adjustments^b^					
		MAF (ILD^+^/ILD^−^)	OR (95% CI)	Crude *P*-value	OR (95% CI)	Adjusted *P*-value	*q*-Value	MAF (ILD^+^/ILD^−^)	OR (95% CI)	Crude *P*-value	OR (95% CI)	Adjusted *P*-value	*q*-Value
rs35705950	T	0.27/0.11	2.81 (1.71, 4.53)	2.8e-05	2.65 (1.59, 4.32)	0.00012	0.0015	0.26/0.11	2.74 (1.65, 4.43)	5.5e-05	2.56 (1.54, 4.21)	0.00023	0.0029
rs111521887	G	0.34/0.20	1.95 (1.30, 2.89)	0.00093	1.90 (1.27, 2.81)	0.0016	0.010	0.34/0.20	2.01 (1.34, 2.98)	0.00060	1.96 (1.31, 2.92)	0.00098	0.0063
rs2609255	G	0.33/0.23	1.63 (1.08, 2.43)	0.017	1.63 (1.07, 2.45)	0.021	0.090	0.32/0.23	1.59 (1.05, 2.38)	0.026	1.61 (1.05, 2.44)	0.027	0.12
rs2736100	A^c^	0.60/0.53	1.36 (0.94, 2.00)	0.11	1.42 (0.97, 2.09)	0.076	0.25	0.60/0.53	1.33 (0.91, 1.95)	0.14	1.38 (0.94, 2.05)	0.10	0.33
rs2076295	G	0.47/0.42	1.25 (0.85, 1.84)	0.25	1.29 (0.88, 1.91)	0.19	0.45	0.46/0.42	1.21 (0.82, 1.77)	0.34	1.25 (0.84, 1.85)	0.26	0.59
rs12610495	G	0.43/0.36	1.33 (0.91, 1.95)	0.14	1.28 (0.87, 1.88)	0.21	0.45	0.42/0.36	1.28 (0.87, 1.88)	0.21	1.22 (0.83, 1.80)	0.31	0.59
rs7887	T	0.48/0.42	1.27 (0.87, 1.86)	0.21	1.18 (0.79, 1.77)	0.42	0.75	0.48/0.42	1.27 (0.87, 1.86)	0.22	1.18 (0.78, 1.77)	0.44	0.71
rs5743890	C	0.09/0.11	0.77 (0.37, 1.43)	0.45	0.78 (0.37, 1.45)	0.46	0.75	0.08/0.11	0.70 (0.33, 1.33)	0.32	0.70 (0.32, 1.34)	0.32	0.59
rs6793295	C	0.33/0.31	1.06 (0.71, 1.57)	0.76	1.11 (0.73, 1.65)	0.61	0.89	0.33/0.32	1.09 (0.72, 1.61)	0.69	1.13 (0.75, 1.68)	0.56	0.82
rs11191865	A	0.44/0.46	0.90 (0.62, 1.32)	0.60	0.93 (0.63, 1.37)	0.73	0.95	0.44/0.46	0.90 (0.61, 1.32)	0.60	0.93 (0.63, 1.38)	0.73	0.87
rs1278769	G	0.22/0.24	0.93 (0.59, 1.42)	0.75	0.94 (0.59, 1.46)	0.80	0.95	0.22/0.24	0.90 (0.56, 1.39)	0.65	0.90 (0.56, 1.40)	0.67	0.87
rs2034650	A	0.49/0.50	0.98 (0.67, 1.44)	0.93	0.99 (0.67, 1.46)	0.96	0.97	0.48/0.50	0.95 (0.64, 1.39)	0.77	0.96 (0.65, 1.42)	0.84	0.91
rs4727443	C	0.36/0.36	0.99 (0.67, 1.46)	0.98	1.01 (0.67, 1.49)	0.97	0.97	0.36/0.36	0.98 (0.66, 1.45)	0.92	1.0 (0.67, 1.48)	1.0	1.0

Only patients with no missing values are included.

a Adjusted for sex, age at RA diagnosis, smoking habits, anti-CCP, RF and HLA-shared epitope.

b Adjusted for sex, age at diagnosis, smoking habits, anti-CCP, RF, HLA-shared epitope and DAS28 at the index date.

c PF-associated allele.

MAF: minor allele frequency.

### Associations with clinical data

The patients affected with PF were significantly older at RA disease onset, were more often RF positive and had higher disease activity during the first 24 months (Table 1). PF was associated with increased risk of death, which remained significant after adjustments for age [adjusted OR 4.55 (95% CI 2.53, 8.16), *P* < 0.001]. Seven of the 11 patients with early diagnosis of PF (within 4 months from the index date) were already being treated with MTX from the index date. Overall, patients selected for MTX treatment were significantly younger, with a mean age of 56.3 years (s.d. 13.4) *vs* 59.5 (16.1) for those not treated with MTX (*P* < 0.001). This was also evident among those with or developing PF; those selected for MTX treatment were significantly younger, with a mean age of 63.7 years (s.d. 8.9) *vs* 67.3 (13.2) for those not treated. However, significantly more patients diagnosed with PF had received bDMARDs already within the first 24 months of disease (20% *vs* 10.8%; *P* < 0.05), irrespective of the time point of the PF diagnosis.

In simple logistic regression analysis, age at onset and positive RF were associated with increased risk of PF, and in multiple variable analysis these factors remained significantly associated with the risk of PF (Table 4). MTX exposure from the index date was associated with a decreased risk of PF [OR 0.21 (95% CI 0.12, 0.39), *P* = 6.0 × 10^−7^), irrespective of smoking and age, while the other variables were associated with increased risk (Table 4). Frequencies of csDMARD treatment during the first 24 months or after 24 months were similar, irrespective of the presence of PF (Table 1).

**Table 4:** Associations between PF and covariates in simple and multivariable analyses

Variables	Simple variable analysis		Multivariable analysis	
	OR (95% CI)	*P*-value	OR (95% CI)	*P*-value
Sex	0.64 (0.35, 1.24)	0.17	0.86 (0.44, 1.74)	0.67
Age at RA onset	1.10 (1.03, 1.09)	2.9e-05	1.10 (1.03, 1.09)	0.0001
Smoking +/−^a^	1.80 (0.92, 3.60)	0.099	1.50 (0.77, 3.21)	0.25
ACPA +/−	1.40 (0.70, 3.01)	0.37	1.20 (0.52, 2.81)	0.73
RF +/−	2.70 (1.10, 7.80)	0.042	3.0 (1.10, 9.58)	0.045
HLA-shared epitope +/−^b^	0.97 (0.51, 2.00)	0.94	0.88 (0.44, 1.89)	0.74
MTX started at index date	0.27 (0.14, 0.51)	4.3e-05	0.30 (0.16, 0.60)	0.00048
BMI^c^	1.0 (0.95, 1.08)	0.64	1.00 (0.95, 1.10)	0.54
DAS28 at index date	0.99 (0.80, 1.22)	0.92	0.91 (0.72, 1.14)	0.39

a Smoking refers to ever being a smoker.

b HLA-shared epitope includes 0401/0404/0405/0408/0101.

c Missing values were imputed.

### Associations between clinical data and SNPs

Including the three SNPs most strongly associated with RA-PF (rs35705950, rs111521887 and rs2609255) in separate multiple variable analyses including the demographic data yielded similar risk ratios for the SNPs for PF. Age, RF positivity and MTX exposure from the index date remained significantly associated with the risk of PF (Table 5). Comparing patients who were carriers of the minor alleles of all three SNPs *vs* those without minor alleles yielded a similar risk for the combination of the three SNPs [OR 2.1 (95% CI 1.4, 3.3), *P* = 0.00049] as did each SNP separately. The other factors (age at RA diagnosis, RF positivity and MTX) remained significant risk factors of the same magnitude in the simple and multiple variable analyses (data not shown).

**Table 5:** Logistic regression analyses with PF as the outcome with the top three SNPs included (rs2609255, rs111521887 and rs35705950) and covariates included as adjustments in previous analyses in multivariable regression models and registered at the index date

Variables	rs 2609255		rs111521887		rs35705950	
	OR (95% CI)	*P*-value	OR (95% CI)	*P*-value	OR (95% CI)	*P*-value
SNP	2.04 (1.22, 3.39)	0.0057	2.19 (1.34, 3.55)	0.0015	2.67 (1.38, 5.02)	0.0026
Sex, female/male	0.80 (0.39, 1.69)	0.54	0.80 (0.39, 1.69)	0.55	0.49 (0.22, 1.09)	0.075
Age at RA diagnosis	1.05 (1.02, 1.08)	0.0030	1.05 (1.02, 1.09)	0.0017	1.05 (1.01, 1.09)	0.0097
Ever-smoker, +/−	2.31 (1.02, 5.92)	0.058	2.30 (1.02, 5.89)	0.060	3.21 (1.17, 11.41)	0.040
BMI, kg^2^/m^a^	1.03 (0.95, 1.11)	0.45	1.04 (0.96, 1.12)	0.32	1.05 (0.95, 1.15)	0.29
DAS28	0.90 (0.71, 1.16)	0.42	0.93 (0.73, 1.19)	0.54	0.84 (0.64, 1.11)	0.23
MTX	0.39 (0.19, 0.87)	0.016	0.38 (0.18, 0.84)	0.013	0.46 (0.18, 1.27)	0.11
ACPA +/−	1.16 (0.48, 3.09)	0.76	1.30 (0.53, 3.54)	0.59	1.57 (0.51, 6.03)	0.47
RF +/−	2.78 (0.94, 10.36)	0.088	2.62 (0.88, 9.84)	0.11	4.05 (1.03, 27.30)	0.080
HLA-shared epitope^b^	0.95 (0.44, 2.22)	0.91	0.81 (0.38, 1.87)	0.61	0.90 (0.36, 2.45)	0.82

a Missing values for BMI are imputed.

b HLA-shared epitope includes 0401/0404/0405/0408/0101.

## Discussion

In this rather small exploratory study of an unselected inception cohort of patients with early RA, associations were found with PF risk gene variants. The PF risk conferred by the *MUC5B* promoter variant rs35705950 was the strongest in our analysis and of the same magnitude in our cohort as in others [16]. Moreover, 3 of the 12 additional genetic loci previously associated with PF in RA were confirmed in our study, including the *TERT* locus when fully adjusted [10, 11, 14]. The remaining nine loci were not associated with PF in RA in our study, possibly due to the limited size of the cohort. We also adjusted for several clinical variables, which was not performed fully in other studies. This study conclusively confirms both a shared genetic predisposition for IPF and RA-associated PF and an RA-specific genetic susceptibility to PF.

Eleven (18.3%) of the RA patients were diagnosed with PF along with the diagnosis of RA, which is a slightly lower frequency than has been found in similar studies, which were 24.7% and 25%, respectively [15, 31]. The reported prevalence in our unselected inception cohort was fairly low, despite the fact that in most cases the diagnosis of PF was based on the results of HRCT, which is a favourable method of detecting fibrosis [32]. The annualized incidence was 5.0/1000 person-years, which is in line with another report of 4.1/1000 person-years [15], although we have focussed on the presence of PF in screening for lung involvement while the other study included ILD [15].

In previously performed studies on IPF as well as in RA-related PF, older age at disease onset, male sex and smoking were predisposing factors [18, 19]. Interestingly, we found that only older age and the presence of RF promoted the development of fibrosis. Male sex and being an ever-smoker were numerically greater in the cases with fibrosis but did not reach statistical significance in simple or multiple variable analyses, concordant with the findings reported for another inception cohort [15]. However, the frequency of being an ever-smoker was, overall, high in our population irrespective of the presence of lung fibrosis, while the frequency of current smokers had decreased to 19% and was similar in both patient groups irrespective of fibrosis. ACPAs were numerically, but not significantly, more frequently positive among the RA patients with fibrosis. This finding is not as convincing as those of a number of studies that reported increased frequencies of ACPAs in RA patients with ILD [5, 19, 21] but is in line with another [33]. In this study focussed on the development of PF, we did not include the development of other lung manifestations, which could explain the lack of a significant association with ACPAs. The frequency of ACPA positivity among patients having or receiving the PF diagnosis when RA was diagnosed was 82%, similar to that in the whole PF group (80%). Taken together, a putative association between the presence of ACPAs and the development of RA-PF remains controversial and larger studies with well-defined cohorts are needed in order to support or reject a common pathogenic mechanism between the two.

Patients with PF had higher disease activity over time, measured as DAS28-AUC_24_, as previously reported [34, 35]. The DAS28 at the index date was similar among patients, irrespective of PF. Further, adjustments for DAS28 in multivariable analyses did not affect the increased risk of PF conveyed by the SNPs. Patients who developed PF were more often treated with bDMARDs already within the first 24 months after the diagnosis of RA, irrespective of diagnosed PF. This suggests, indirectly, that they had a higher disease activity. Also, only 4 of the 12 cases treated with bDMARDs within the first 24 months were diagnosed with PF during that period. csDMARDs were used to the same extent as for those without fibrosis—in 98% of the cases—although to a significantly lesser extent with MTX from baseline.

Although this study was not designed to analyse the relationship between MTX treatment and PF in depth, we could clearly see that MTX exposure from the index date was associated with a significantly reduced risk of fibrosis. These findings are in line with a previous publication of a reduced incidence of RA-ILD in patients treated with MTX [31]. In another study, no supporting evidence was presented for a relationship between MTX and RA-ILD [15], and in a recent review of the literature, firm conclusions about an association between MTX and ILD could not be drawn [36]. Another recent nationwide population-based study found no increased risk of MTX treatment for RA-ILD [37]. The choice of pharmacological treatment for individual RA patients in our cohort was made by the patient’s physician in agreement with the patient, based on the Swedish national guidelines. Notably, in our study, patients diagnosed with PF were less often already being treated with MTX from baseline and also were less often treated with MTX when the fibrosis was diagnosed at a later stage during disease progression. Patients selected for MTX treatment were significantly younger than patients who started other treatments. One possible explanation for this could be that at the time of RA diagnosis, older individuals present with more comorbidities or other factors that would drive the selection of treatments towards other csDMARDs. Thus we suspect, but have not been able to show in our available data, that absolute or relative contraindications for MTX coincided with or were associated with risk factors for PF, such as older age, causing MTX to less often be the choice for patients who later developed PF.

The strength of the current study is that the cohort of RA patients was unselected and originated from a homogeneous population of northern Sweden. Almost all (95%) individuals diagnosed with early RA within the catchment area of northern Sweden and included in the SRQ were included and willing to participate in the study. Furthermore, X-rays of the lungs of the patients were routinely performed at inclusion, providing baseline information about the lungs.

A limitation of the study is that the HRCT examinations were not performed randomly or on all included patients, but for those with abnormalities on the plain X-rays or clinical indications with the development of defined symptoms. The HRCT examinations have been performed over a period of almost 20 years and methodological improvements during this time could affect the results. Consequently we refrained from further diagnostic evaluations besides PF. Another limitation is that the controls were identified within the population-based biobank of northern Sweden and we lack information about any comorbidities among the them. However, the potential prevalence of PF among the controls would reduce the contrast in the comparison with our affected cases.

From this study we can conclude that RA-related factors with the exceptions of age at diagnosis and positivity for RF have limited importance compared with the impact of ILD-associated genetic factors in the development of PF in RA. These findings would support the use of the newly introduced antifibrotic targeted therapies in the treatment of patients with RA and PF to prevent further fibrotic development.
